# Assessment of the Possibility of Using Unmanned Aerial Vehicles (UAVs) for the Documentation of Hiking Trails in Alpine Areas

**DOI:** 10.3390/s18010081

**Published:** 2017-12-29

**Authors:** Paweł Ćwiąkała, Rafał Kocierz, Edyta Puniach, Michał Nędzka, Karolina Mamczarz, Witold Niewiem, Paweł Wiącek

**Affiliations:** AGH University of Science and Technology, Faculty of Mining Surveying and Environmental Engineering, 30-059 Cracow, Poland; pawelcwi@agh.edu.pl (P.Ć.); kocierz@agh.edu.pl (R.K.); mnedzka@agh.edu.pl (M.N.); karolina.mamczarz@op.pl (K.M.); w.niewiem@gmail.com (W.N.); paw.wiacek@gmail.com (P.W.)

**Keywords:** UAV, monitoring, mountain trails

## Abstract

The research described in this paper deals with the documentation of hiking trails in alpine areas. The study presents a novel research topic, applying up-to-date survey techniques and top quality equipment with practical applications in nature conservation. The research presents the initial part of the process—capturing imagery, photogrammetric processing, quality checking, and a discussion on possibilities of the further data analysis. The research described in this article was conducted in the Tatra National Park (TNP) in Poland, which is considered as one of the most-visited national parks in Europe. The exceptional popularity of this place is responsible for intensification of morphogenetic processes, resulting in the development of numerous forms of erosion. This article presents the outcomes of research, whose purpose was to verify the usability of UAVs to check the condition of hiking trails in alpine areas. An octocopter equipped with a non-metric camera was used for measurements. Unlike traditional methods of measuring landscape features, such a solution facilitates acquisition of quasi-continuous data that has uniform resolution throughout the study area and high spatial accuracy. It is also a relatively cheap technology, which is its main advantage over equally popular laser scanning. The paper presents the complete methodology of data acquisition in harsh conditions and demanding locations of hiking trails on steep Tatra slopes. The paper also describes stages that lead to the elaboration of basic photogrammetric products relying on structure from motion (SfM) technology and evaluates the accuracy of the materials obtained. Finally, it shows the applicability of the prepared products to the evaluation of the spatial reach and intensity of erosion along hiking trails, and to the study of plant succession or tree stand condition in the area located next to hiking trails.

## 1. Introduction

Alpine areas are shaped by interacting morphogenetic processes [1,2]. Their activity depends on local environmental conditions, such as geological structure, relief, topoclimate, and the spatial reach of altitudinal zones [1]. Avalanches [3,4], landslides [5], and debris flows [6] are typical processes in alpine areas. They are the main cause of transformation of mountainous area landscapes in the world [7,8]. The Tatras slopes are being modelled by debris flows, mud-debris flows, and torrential flows, also by soil material flushing, rill erosion, nivation, solifluction, creep, aeolian processes, and the effects caused by needle ice [1,9]. These processes transform the Tatra slopes with varying intensity and their effects are differentiated in time and space.

An additional factor that increases the activity of erosive processes in high mountains is hiking [10,11,12]. The anthropogenic degradation on hiking trails on slopes is currently the subject of research, both in Poland [13,14] and worldwide [15,16,17].

In order to determine the effects of anthropogenic degradation on hiking trails it is necessary to examine the tourist flow and its spatial and temporal distribution. To this end, different methods were used. They include counting the number of tourists by people trained for this purpose [12], registration of tourists using the video system [18] or the use of pyroelectric sensors to accurately determine the distribution of tourist movements [19].

The effects of tourism are easily visible in the relief of hiking trails and paths and appear as various landforms created by erosion, denudation, or accumulation processes. Traditional methods of relief measurements include geomorphological mapping at various scales, test points, and surveys in slope catenas. The dynamics of the processes is typically determined with simple measurement techniques, such as a line of pegs driven into the ground at different depths, lines of marked debris to measure cover movements, nets to catch material falling off the rock walls, and instruments to measure the size of lifting [20]. Currently, surveying methods are used in topography. They cover traditional measurement techniques, such as satellite measurements [21] or total stations. They supply accurate and discrete data about the relief. Unfortunately, they are time-consuming and, therefore, they are used for small areas. In turn, remote sensing methods, such as terrestrial (TLS) and airborne laser scanning (ALS) [22,23], aerial and terrestrial photogrammetry [24,25], and satellite imaging, enable surveying large areas and provide quasi-continuous data. Their drawback is their high price. Despite this fact, they are increasingly being used in geomorphology and facilitate the acquisition of rich collections of data, with varying precision and detail.

Recently, UAVs, which are used to obtain spatial data, have become quite popular. Currently, the most common sensors installed on UAVs are non-metric cameras. However, devices equipped with infrared cameras, multi-spectral cameras, or light detection and ranging (LIDAR) scanners are becoming more and more popular [26,27]. Depending on the type of the flying unit, the UAV enables performing measurements over the areas ranging from a few acres to several square kilometres [28,29,30]. It is worth noting that this new and very popular method of spatial data acquisition ensures the required measurement accuracy. Ground sampling distance (GSD) is of crucial importance here. It depends mainly on the flight level and the camera sensors and lenses used to take measurements. The second important aspect is to provide the final products with georeference data. Currently, mainly ground points measured with traditional survey techniques are used for this purpose. There are also other solutions, such as the use of point coordinates obtained from other sources, for example, from satellite images [28]. Georeference information can be also supplied directly by measuring exact coordinates of images’ projection centres. Such solutions are typically used in situations when the UAV is fitted with a satellite receiver which determines the UAV position in real time, and with inertial navigation systems (INS) [31,32].

Another factor which affects the accuracy of results obtained from the UAV data is topography of the area, which is the subject of this research. Most publications indicate that products generated by UAV flights are verified with satellite measurement methods (in the kinematic or static mode), tacheometry, and TLS. It is worth noting that, in many cases, both the products obtained from UAV and TLS data are burdened with the influence of vegetation, which negatively affects the quality of both datasets (from the UAV and TLS) [33]. Therefore, TLS is not always a reliable method for referential data acquisition. Taking this into account, analyses that rely on independent classical measurements (total station, satellite measurements) should be considered as more reliable [34]. In the areas where the landscape is not very diversified (flat areas) [35,36] the accuracy of the final products approximate 2–3 GSD [29,37]. However, according to our best knowledge, no studies have been conducted so far to determine the accuracy of UAV-based photogrammetric products in areas of such large height differences. The relative height differences of the area being subject to the tests described in this article are up to 1000 m.

Despite the fact that photogrammetric measurements taken from low levels have lower accuracies than the data obtained from TLS, their great advantages include even distribution of survey points in the study area, less effort needed to acquire data, and a lower price of the equipment. Considering the products generated during UAV flights (digital elevation models (DEM), orthophotomosaics), UAVs may be widely applied to monitor landslides [34,38,39,40,41], soil and slope erosion [29,42,43], and to measure seashores in order to check the evolution of beaches and cliffs [44,45,46]. It is worth noting that one may come across publications that suggest using UAVs for periodic monitoring of landslides and TLS for measuring large deformations of objects that occur after extreme events [41]. UAV surveys also find applications in vegetation monitoring [47], precision agriculture enhancement [48], information acquisition concerning damage caused by natural disasters [49,50], geological surveys [51], and visual investigation of large-scale structures, such as dams [52].

There are a variety of photogrammetric software tools available for UAV-based data. The use of web services and software packages that “automatically generate 3D points from arbitrary image configurations” was examined by Neitzel and Klonowski [53]. Vallet et al. [54] compared georeferenced DTMs produced from Pix4D and NGATE (in SOCET SET). The objective of the study presented in [55] is to investigate the level of accuracy that can be achieved using two of these software tools: Agisoft PhotoScan Pro and an open-source alternative, IGN MicMac, in sub-optimal survey conditions. On the basis of these studies, it can be concluded that the applied software influences the obtained results.

The most important aim of the research described in this article was to document the selected linear hiking trails in the Western Tatras between the lower subalpine zones and alpine glades. The current state of the trails was established using the photogrammetric method (UAV), whose accuracy was verified with other surveying methods (measurement of control profiles, TLS). The data obtained, after comparing them with archival data provided by the TNP (or data obtained in successive measurement series) comprise valuable information which enables, for example, the examination of the trails’ erosion degree, the progress of forest succession, and the impact of anthropogenic denudation on tree line changes.

The outcome of the work comprise digital surface models (DSM) and/or digital terrain models (DTM) in two periods: primary and secondary. The current model was created on the basis of data obtained with the use of a UAV. The data for the creation of the original model came from ALS and was supplied by the TNP. It is worth noting that in the longer perspective, the follow-up measurements in the same area will facilitate a more in-depth analysis of the impact that tourism has on the environment.

## 2. Materials and Methods

### 2.1. Study Site

**Figure 1:**
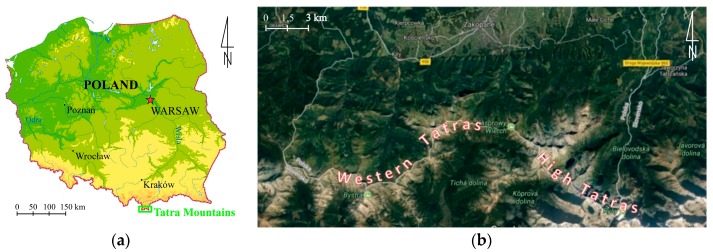
Location of the study area: (**a**) location of the Tatra Mountains in Poland, (**b**) location of the Western Tatras and High Tatras (source: own study based on [60]).

Research described in this article was conducted in the Tatra National Park, which is situated in the southern part of Poland (Figure 1). The Tatras comprise the only alpine area in Poland. Due to outstanding natural features of this area, the national park was founded here in 1955. It is one of the largest national parks in Poland with the total area of 21,197 ha [56]. In comparison to other national parks in Europe it is a relatively small park. 

The TNP is one of the most visited parks in Poland. It is visited by 3.5 million tourists annually [56]. It is also one of the most visited parks in Europe. In comparison, alpine parks are visited on average by 700,000 tourists every year [57]. Although the density of hiking trails in TNP is 1.3 km/km^2^ and is much higher than density of alpine and Pyrenean parks, a serious problem in the Tatras is the number of tourists, which exceeds the capacity of hiking trails [58]. The exceptional popularity of this place causes the destruction of vegetation and soil cover and contributes to the reinforcement of the morphogenetic processes, resulting in the development of numerous forms of erosion. The issue of excessive tourism in the Tatras and its impact on the landscape transformation has been widely discussed in the literature [12,14,57,58,59].

**Figure 2:**
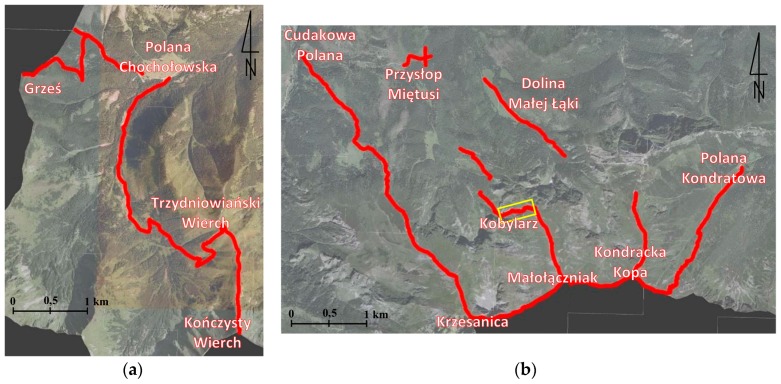
Hiking trails in the Western Tatras which are the subject of this research: (**a**) western part of the research area, (**b**) eastern part of the research area (source: own study based on [61]).

The linear hiking trails in the Western Tatras were used as case studies to verify whether it is possible to use a UAV to evaluate the spatial reach and intensity of erosion in the alpine areas. It has also been verified how accurately can detect relief changes by this method. In total, about 27 kilometres of hiking trails were measured (Figure 2), which constitutes about 10% of all trails in the TNP. Some of these trails were overhauled recently (2014–2015). Their total length is 13.8 km. The length of the trails which have not been repaired is 13.2 km.

### 2.2. Data Acquisition

**Figure 3:**
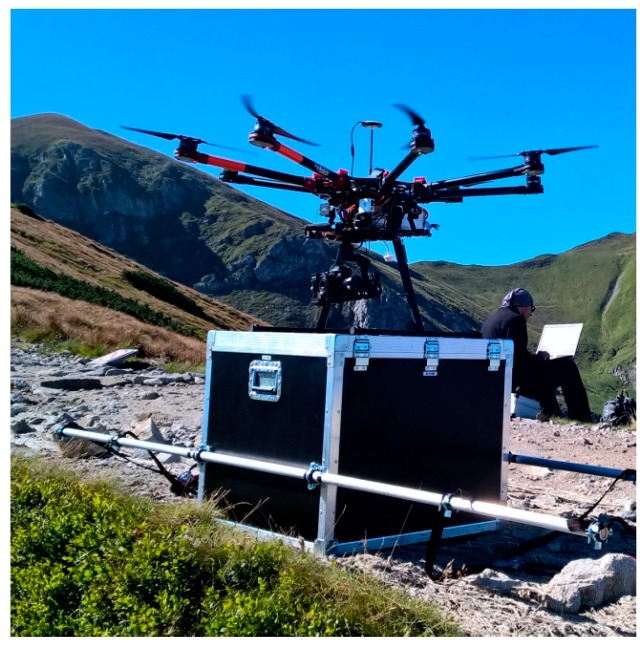
The device used for measurements, the DJI S1000 multicopter (source: authors’ own study).

For data acquisition, a model S1000+ UAV manufactured by the DJI Company (Shenzhen, China), (Figure 3) was used. It is an octocopter equipped with the DJI A2 flight controller with a GNSS receiver, a compass, and IMU, which enable the determination of the device’s horizontal position with an accuracy of ±1.5 m, and height with the accuracy of ±0.5 m. According to information provided by the manufacturer, the device works properly when the wind does not exceed 8 m/s and the inclination is not more than 35° [62]. The UAV was fitted with a Alfa A7R camera (Sony, Tokyo, Japan) equipped with a Sony Zeiss Sonnar T* FE 35 mm F2,8 ZA lens, whose position was stabilised with a Zenmuse Z15-A7 gimbal (DJI, Shenzhen, China).

Measurements were carried out in August and September 2016. They included registration of vertical images of trails from onboard the UAV along with georeference information. All UAV flights were carried out by the holders of Unmanned Aerial Vehicle Operator (UAVO) qualifications certificate, while maintaining high security standards in the Visual Line of Sight (VLOS) conditions.

Field surveys with the UAV were preceded by drawing up UAV flight plans for each hiking trail which was subject of the research. It was assumed that the GSD would be:

- 15 mm in non-forested areas; and
- 20 mm in the areas covered with high trees.

Taking into account features of the equipment used for measurements and the characteristics of the study area, the following assumptions were made:

- the UAV real flight height (above ground level) over the non-forested areas was 100 m;
- the UAV real flight height (above ground level) over the forested areas was 150 m;
- the minimum side overlap between images in adjacent strips was 70%; and
- the minimum forward overlap between images in the strip was 85%.

**Figure 4:**
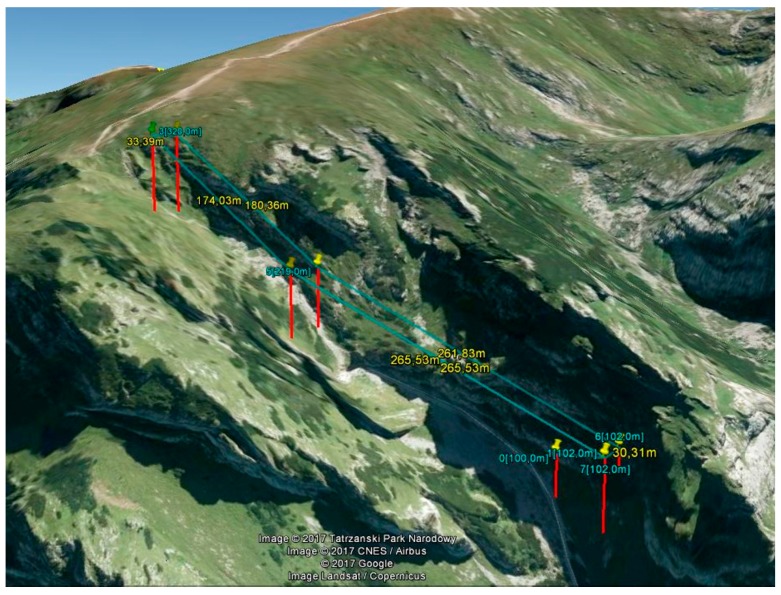
The UAV mission for a section of the trail from the Przysłop Miętusi glade to Małołączniak in the area of Kobylarzowy Żleb (source: authors’ own study). The location of the mission area is indicated by a yellow rectangle in Figure 2b.

The Tatras’ steep slopes were a very demanding study area, so the DTM that was created on the basis of the ALS point cloud was used to plan the UAV flights. Therefore, flight plans considered the height differences along the trail, which exceeded 1000 m. For each hiking trail UAV flights were planned in the two parallel strips which were also parallel to the trail axis. In this way 37 flights were planned with lengths ranging from 0.7 km to 3.2 km. They were drawn up in the DJI Ground Station software. The example of the planned flight mission is shown in Figure 4. The real flight height above ground level and the maximum height differences between waypoints in each UAV flight are presented in Table 1.

The missions’ projects also included the optimal take-off and landing sites. They had to be easily accessible from the trail, far from obstructions, such as high trees or rock walls, and it was also important to ensure that during the UAV flight mission the device could not be present below the start point height.

It should be emphasized that the planning of UAV flights over difficult terrain, which is a very steep slope of alpine mountains, requires great care and precision. This problem was highlighted in [63]. In that paper, the authors also pointed out the need for accurate flight planning in order to maintain the desired GSD. Resolving this problem for difficult alpine areas is described in detail above.

**Figure 5:**
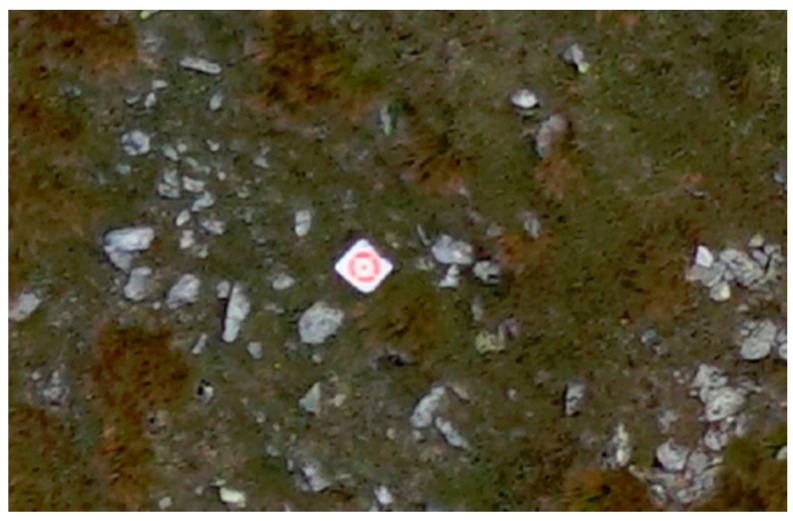
A control point on the image taken from the UAV (source: authors’ own study).

During the field works, before each planned UAV flight, it was necessary to set out, mark, and measure coordinates of control points and check points (Figure 5) in the area covered by the UAV flight. Previously, however, the project of the photogrammetric control point distribution (control points and check points) along the UAV flight routes was made. It was assumed that pairs of points should be located (on both sides of the trail) in the interval of approximately 100 m. The correctness of the assumption was verified by analysing how the number and distribution of ground control points affect the accuracy of the DSM/DTM of the elongated areas, which is the subject of a separate publication [64]. Due to difficult terrain conditions, the project was also verified on the basis of orthophotomap and DTM provided by the TNP (archival aerial orthophotomap and archival DTM from ALS data), in order to evade wooded areas (lack of visibility from the air) and areas which are inaccessible due to the steepness of the slopes.

Control points and check points were measured with the real-time network (RTN) GNSS method referenced to a network of reference stations NadowskiNET or the real-time kinematic (RTK) GNSS method referenced to one of ten base stations that were distributed evenly within the study area.

In the case of RTN GNSS measurements, the final coordinates were immediately calculated in the field and registered in the controller. The primary problem for the RTK GNSS method (using our own base station) was to determine the spatial coordinates of the base station. To this end, throughout the entire period of kinematic measurements the phase observations were registered on the base stations. These observations along with data from NTW1 (Nowy Targ) reference station, which belongs to the ASG-EUPOS system were used to determine coordinates of the base stations with the accuracy not lower than 1 cm. Based on the coordinates of the base stations, final coordinates of points were calculated. In total, 537 points were measured in the PL-2000/7 planar coordinate system (PL-ETRF2000) and Krondstat86 height system (PL-KRON86-NH) based on the levelling quasi geoid PL-geoid-2011. The accuracy of obtained coordinates is at the level of 2–3 cm.

### 2.3. Data Processing

Image processing was conducted in Agisoft PhotoScan Professional software (Agisoft LLC, St. Petersburg, Russia) which employs full SfM multi-view stereopsis and estimates internal optical parameters and spatial positioning of the respective cameras. Due to the large size of UAV data, it was also necessary to use appropriate hardware. The computer with two Intel Xeon E5 processors, 64 GB RAM, and an Nvidia Quadro K4200 graphics card was used for the photogrammetric processing.

The first step of image processing was to align the images. At this stage, the images were uploaded to the software and were given the initial orientation by adding approximate coordinates of images’ projection centres. In total, 7111 images were used. Images were combined in pairs automatically with the limit of 4000 tie points per pair. Images were aligned when parameters that analyse the image are at the level of single pixels. After images had been aligned, control points and check points were indicated on the individual images. This was preceded by uploading coordinates of the terrestrial photogrammetric control to the software. Each marker was indicated on all photos where it was visible. Table 1 shows a list of the regions (photogrammetric missions combined in groups) along with the number of images, as well as control points and check points that were used for alignment.

With the block of photographs prepared in such a way for each mission, the initial alignment commenced. At this stage, some of points of the photogrammetric control worked as control points and the others as check points, which made it possible to evaluate the project accuracy. The block of photographs was aligned and, at the same time, the camera calibration parameters were determined. In this process the following values were determined: the principal distance (c), the location of the principal point (*c_x_* and *c_y_*) and distortion parameters (*k_1_*, *k_2_*, *k_3_*, and *p_1_* and *p_2_*). As a result of the alignment the root mean square errors of the control points and check points were obtained. They are listed in Table 2. On this basis the accuracy of the generated products was initially assessed.

Then, the final alignment (optimization) of blocks of photographs was performed. This process involved all points of the photogrammetric control (both control points and check points). The final mean square errors of these points coordinates are listed in Table 3. Mean values for these parameters calculated for all the missions are: *m*_x_ = 29 mm, *m*_y_ = 29 mm, and *m*_h_ = 31 mm, which correspond to the error of the horizontal point position *m*_xy_ = 41 mm and the error of spatial position *m*_xyh_ = 51 mm. The worst results of RMSE errors were obtained for the missions in the Kobylarz region. This was due to a small number of control points, which were difficult to set up due to field conditions (steep slopes and exposure).

The next step was to create a dense point cloud with the method of dense matching. As a result, a total of 7,854,760,000 points were generated in all missions. Table 4 shows the size of point clouds for each region. In addition, a number of tie points, which were generated when stitching images, was given. After creating a point cloud for each mission, point clouds were connected in larger groups. The aim of this step was to generate the largest study areas possible in coherent blocks. Then DSMs were created. These were used as a basis to conduct orthorectification of images and to create orthophotomaps. In the end, the finished products (point clouds, orthophotomaps, DSMs) were exported. Table 4 shows the resolution of the final products. It is important to note that before the ultimate generation of an orthophotomap, it was necessary to deprive it of all artifacts, such as people who move.

### 2.4. Control Measurements

To verify the accuracy of the remotely-sensed data, TLS measurements of selected sections of trails and measurements of control profiles in various places along the trails were conducted.

**Figure 6:**
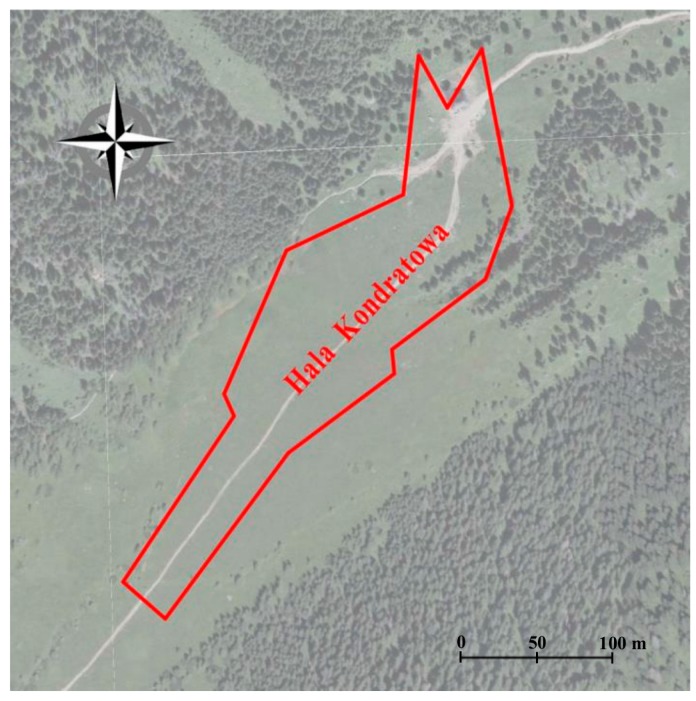
Location and spatial reach of the area where TLS was carried out (Hala Kondratowa) (source: authors’ own study based on [61]).

The TLS measurements covered a 400-metre section of the trail on Hala Kondratowa (Figure 6). A Leica ScanStation C10 laser scanner (Leica Geosystems, St. Gallen, Switzerland) and the set of Leica HDS 6″ targets were used for measurements. The point clouds were registered with the traverse technique based on the three tripods method. Measurements were taken on 16 stations in the strip of land whose width was about 60 m. Each point cloud on the station was registered with the resolution of 10 mm/10 m. Spatial coordinates of 15 scanner stations were set with RTK GNSS measurements with the accuracy of ±1.5 cm. This enabled to significantly reduce propagation of errors between the scanner stations. The TLS data was processed with the Leica Cyclone software. At the beginning, the scanning traverse was aligned in the local coordinate system and the obtained data was merged into one point cloud. Then, the point cloud was transformed into the PL-2000/7 system and the final alignment was performed. The mean transformation error was 0.032 m. The next stage of work was clearing the point cloud of points representing, inter alia, people on the trail, surveying equipment, and artificial landscape features. Manual removal of the non-corrected data was carried out. In the end, data was filtered and the resulting point cloud was manually divided into two layers: ground and trees.

In order to validate the accuracy of the DSM obtained from UAV-borne photogrammetry, cross-sections of trails were also measured within the study area. Measurements were taken by means of RTK GNSS or RTN GNSS methods. The measurement methodology was analogous to the one applied for the photogrammetric control. Control profiles were measured in such places as Małołączniak, Przełęcz Kondracka, along Dolina Kondratowa, along the trail at Ratuszowe Źródło (Czerwony Grzbiet), along the trail from Cudakowa Polana to Ciemniak.

## 3. Results and Discussion

### 3.1. Accuracy Analysis of Products Obtained from UAV-Borne Photogrammetry

As a result of image processing in Agisoft Photoscan Professional software (Agisoft LLC), the RMSE of the spatial position of control points and check points obtained during the initial alignment of the block of photographs are 41 mm and 60 mm, respectively (Table 2). Ultimately, for all the points of the photogrammetric control this parameter amounted to 51 mm (Table 3). These are the values which only indicate the possible accuracies of the products created. It is worth noting that the maximum errors *m*_xyh_ for control points and check points were over 100 mm and were obtained for Kobylarzowy Żleb. This is a particularly difficult area because of the significant height difference exceeding 300 m on the trail section of about 500 m. For other areas the analysed parameter did not exceed 50 mm.

In many publications, the evaluation of accuracy of products obtained from UAV-borne photogrammeters is based only on RMSE analysis for check points. The authors of the paper [65] conducted a study of UAV usefulness for documenting landslides. Based on the results of the study, it was found that applying UAV and SfM enabled to create DSMs of the landslide surface with an accuracy of 4–5 cm in the horizontal and 3–4 cm in the vertical direction. The results obtained are similar to these presented in this paper, but are related to a small object with a slight height differences. However, in [63] the presented tests were conducted on an elongated object, but it was an area with a small inclination along the axis of the UAV flight. The flight plan was a single strip only. The accuracy of the orthophotomosaic was based on a model with only eight control points and 50 check points. The comparison between the coordinates of points extracted from the UAV orthophotomap and their counterparts obtained with the GNSS receiver (i.e., check points) showed a RMSE of 58 and 56 mm for coordinates X and Y, respectively. Our research, similarly as in the above mentioned paper, concerns elongated objects, but with a much more complicated relief. Our results enabled to determine the accuracy and usefulness of UAV data obtained for steep mountain trails for determining even minor changes in their relief. In order to perform a reliable assessment of the accuracy of the products obtained from UAV-borne photogrammetry, control measurements were additionally made (described under Section 2.4).

#### 3.1.1. Evaluation of the Accuracy Based on TLS Data

Data obtained from TLS is of high accuracy and resolution, and the resolution varies substantially and depends on the distance from the station. These characteristics have determined that the TLS method is widely used to evaluate the accuracy of the products obtained from UAV-borne photogrammetry [33,35,55]. Unfortunately, the results of such comparisons do not always give reliable results to assess the accuracy of UAV-based photogrammetric products. In [55] mean differences between the photogrammetric DSMs and the TLS point cloud lower than 20 cm are achieved and standard deviations renged from 0.7 m to 1.1 m (depending on the software used). However, it should be noted that the offsets between the photogrammetric DSMs and the TLS point cloud were low within the control region. Deviations appear outside of the control region, growing with distance to the control region.

**Figure 7:**
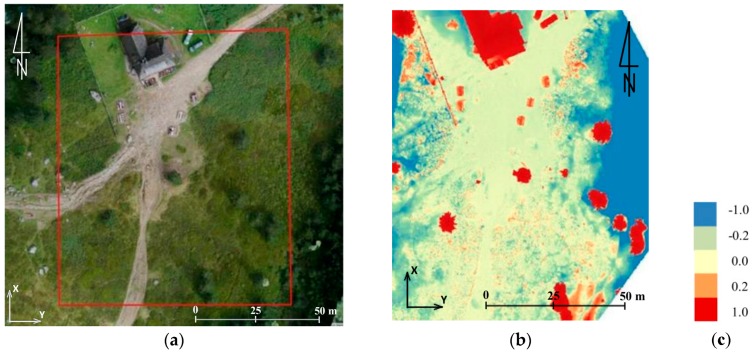
The shelter on Hala Kondratowa: a comparison of DTM from TLS with DSM from UAV: (**a**) the study area, (**b**) results of comparison, and (**c**) colour scale (the units given in m) (source: authors’ own study).

In this paper in the first step of accuracy assessment DTM obtained from TLS data were compared with of the DSM that was generated from UAV-borne photogrammetry. Data analysis was based on the comparison of raster layers. It was for two reasons. First, analyses of this type require much less processing power, which is of great importance when handling data which has a very high spatial resolution. Secondly, the input data was characterized by similar spatial resolution (3 cm). Therefore, before the analyses could start, TLS point clouds were cleared off manually to remove objects of the land cover and then the open source project CloudCompare software was used to generate rasters whose resolution was 3 cm. Further calculations were done in QGIS (open source project) and results were presented in bitmap form (Figure 7).

By comparing data from TLS, and data obtained from the UAV, it was possible to estimate the accuracy of the results obtained. The example presented in Figure 7, enables to draw the following conclusions:

- data within the trail area not covered with vegetation feature high compliance (around 5 cm),
- significant differences may be noticed on objects of the land cover; they result from comparing DTM-TLS and DSM-UAV,
- large differences on the border of the study area are caused by interpolation of data from TLS and small scanning resolution in the distance above 30 m from the trail.

The problem of elimination of the influence of vegetation on the results of comparison of the TLS data and the UAV-based product was discussed, among others, in [66]. Studies presented in this paper were carried out on a landslide which extends over a horizontal distance of 850 m between elevations of 2105 m at the crown, and 1740 m at the toe, with an average slope of 25°. The quality of the photogrammetric DTM was assessed by subtracting the overlapping TLS DTM. In the vertical direction the RMS difference was 0.31 m although maximum deviations reached +3.44 to −4.08 m. The authors pointed out that the most significant errors are induced by some small trees and bushes, the effects of which could not be reliably removed from the photogrammetric DTM. The same conclusion was formulated in [33]. The article compared the data from the UAV equipped with RGB camera with TLS and total station measurements. The authors also presented a procedure of filtering point clouds, the results of which allow obtaining point clouds representing DTM without data representing vegetation. Application of filtration significantly reduced the errors.

**Figure 8:**
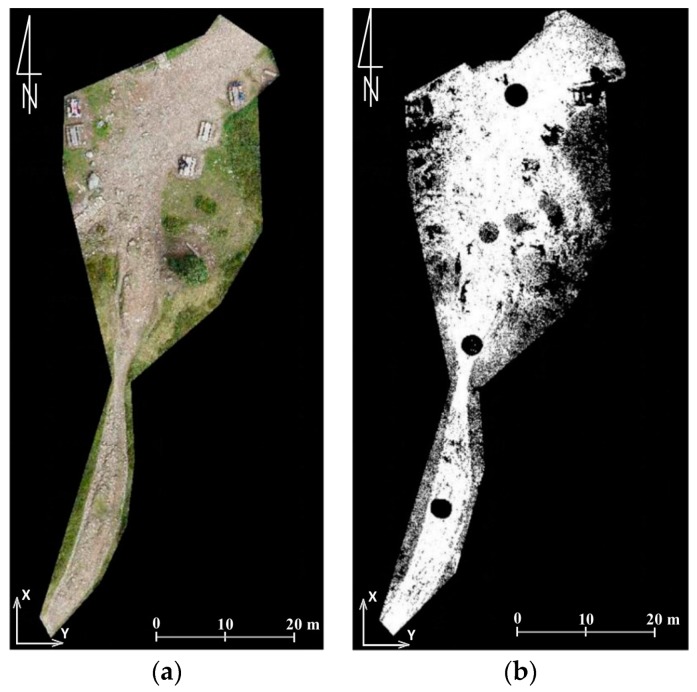
Point clouds from the UAV (**a**) and TLS (**b**) which represent the first test field (source: authors’ own study).

**Figure 9:**
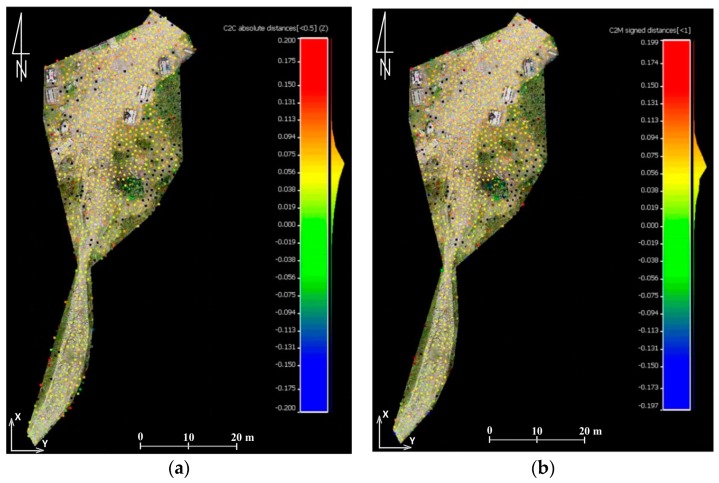
Results of comparing the point cloud from TLS with the point cloud from the UAV (**a**) and mesh model from the UAV (**b**) in certain points for the first test field (source: authors’ own study) (the units given are in m).

**Figure 10:**
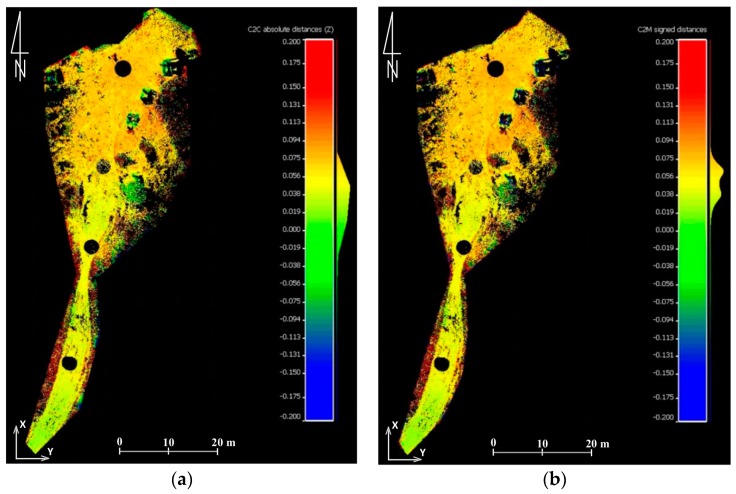
Results of comparing the point cloud from TLS with the point cloud from the UAV (**a**) and mesh model from the UAV (**b**) for the first test field (source: authors’ own study) (the units given in m).

**Figure 11:**
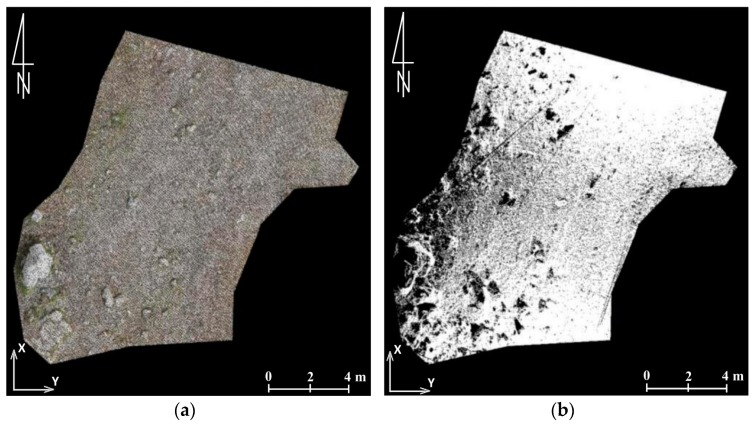

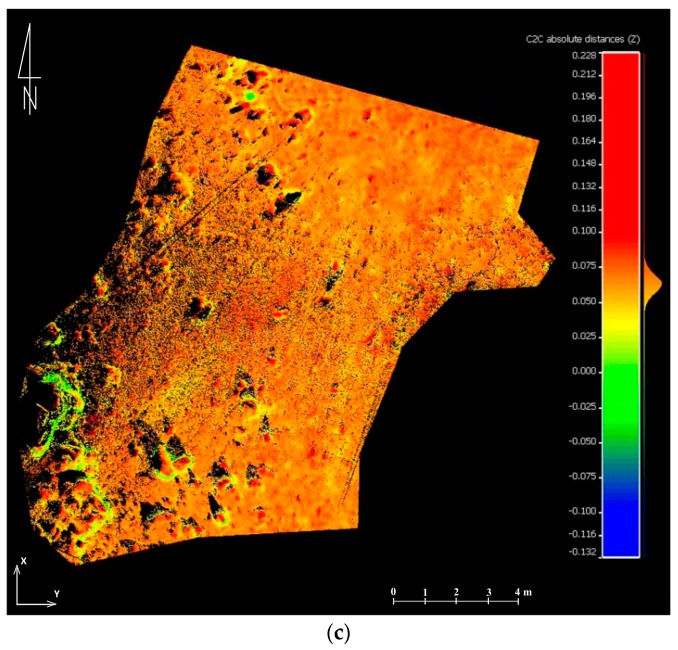
Point clouds from the UAV (**a**) and TLS (**b**) which represent the second test field and results of their comparison (**c**) (source: authors’ own study) (the units given in m).

In order to obtain figures that describe the accuracy of data obtained from the UAV only on hiking trails, which was the subject of research described in this article, the authors also conducted analyses that compare the point cloud from TLS with the point cloud and mesh generated from the UAV pictures. Two test fields were identified (Figure 8 and Figure 11). The first one covered the section of the trail by the shelter on Hala Kondratowa and the surrounding area. In the first step differences between data from the UAV and data from TLS were analysed in certain points. For this reason, a point cloud from TLS was thinned to the mesh with a cell size of 1 m and compared with a point cloud and mesh from the UAV (Figure 9). The average difference was about 0.05 m in both cases and standard deviation was around 0.05 m (Table 5). Similar calculations were done for the whole set of data obtained from TLS (Figure 10). In this case the average differences slightly exceeded 0.04 m (standard deviation was around 0.04 m) (Table 5).

The second area covers the part of the first area with a fragment of the trail in the immediate vicinity of the shelter on Hala Kondratowa. It is characterised by the lack of plants and a high density of points from TLS. For this area, differences between the point cloud from TLS and the point cloud generated from the UAV images were determined. For this area the average difference was 0.062 m and standard deviation came to 0.013 m (Table 5).

The low standard deviation obtained from the second test field (Table 5, Figure 11) indicates that both methods projected the relief in a similar way and the greatest differences occurred on the rock borders. The higher deviation values for the first test field result from different densities of the two sets of points and vegetation that appears in this area. The average distance value suggests that there is a difference between the methods. It may be caused by the accuracy with which georeference data is supplied or by not maintaining the scale parameter and it indicates accuracy level, at which these two methods can be compared.

Although TLS-based point clouds are as dense as the UAV-based point clouds, TLS data are subject to shadowing due to the oblique view point, what is shown in Figure 8, Figure 9, Figure 10 and Figure 11. Such shadows are minimised in nadir UAV-acquired images and large-scale data acquisition can be obtained more effectively by UAV. The same conclusions were drawn in [66].

#### 3.1.2. Evaluation of the Accuracy Based on the Control Profile Measurements

The accuracy of UAV-based products were also evaluated on the basis of control profiles. A similar solution was implemented in the studies presented in [67], but on a much smaller scale and without a detailed analysis of the results.

The points on the control profiles were measured by the RTK GNSS or RTN GNSS method. The method of measuring and calculating the coordinates of these points was the same as for the points of the photogrammetric control. For each control profile height differences between points in the profile recorded with different measurement methods were analysed. The authors used all types of information on relief that was available, that is:

- points measured with RTK/RTN GNSS methods: data of highest spatial accuracy (±2–3 cm), but also of low resolution. It is important to note the problem of relief generalisation that appears during discrete measurements;
- DTM acquired from TLS: data of high spatial accuracy (±5 cm) and of very high resolution (3 cm) along the trail;
- DSM acquired from the UAV: data of high spatial accuracy (±5 cm) and of very high resolution (3–5 cm); and
- DTM generated from the archival ALS points cloud: data of the lowest spatial accuracy (±30 cm) and of low resolution (35 cm).

**Figure 12:**
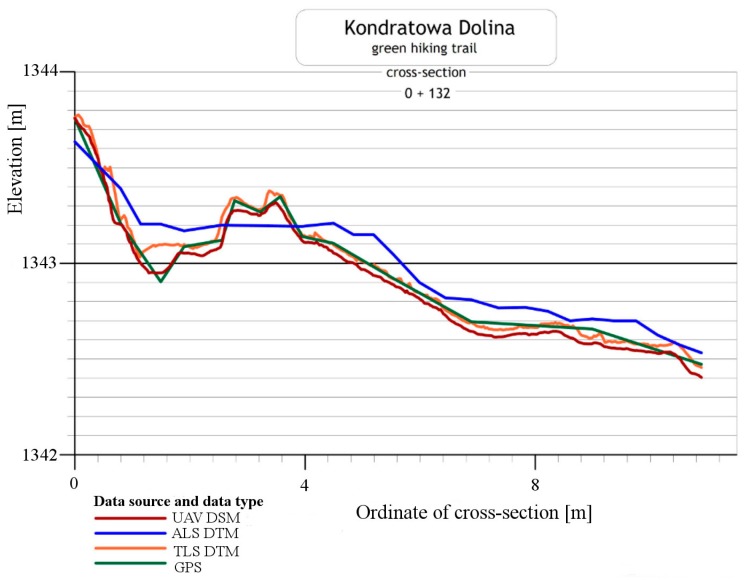
Example of the cross-section of the hiking trails determined with different survey methods (UAV, ALS, TLS, GPS) (source: authors’ own study).

Figure 12 presents an example of the cross-section. Although RTK/RTN GNSS measurements feature low density, it is easy to notice a very high compatibility of heights obtained with the model generated on the basis of the UAV images. This confirms the fact that both measurements were performed correctly. On the other hand, UAV DSM has lower elevations compared to other DTMs (TLS DTM and ALS DTM). In general, DSM should have higher elevation compared to DTM. Significant differences between UAV data and data from ALS (approx. 30 cm) result from the accuracy of this type of data. It must be emphasised that data from ALS comes from 2012. Therefore, it is necessary to consider the impact the small relief changes may have on the presented outcome.

In the next part of the study, RTK/RTN GNSS method was recognised as the most accurate one and the authors of this paper identified parameters which enable the evaluation of the accuracy of the results generated with other methods, i.e., the mean height difference, maximum and minimum difference, and standard deviation of differences (Table 6 and Table 7). The analysis was conducted using two scenarios. The first scenario considered all points measured with the RTK/RTN GNSS technique (points on the trail and points off the trail in the distance of up to 20 m) whereas, in the second scenario, only the points on the paved section of the trail were measured.

**Figure 13:**
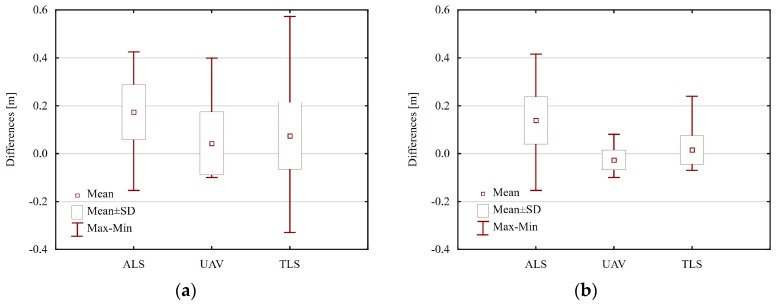
Box plot presenting parameters of accuracy evaluation for data obtained from ALS, UAV, and TLS against the RTK/RTN GNSS measurements: (**a**) for points located on trails and in their immediate vicinity; and (**b**) for points located on the trails (source: authors’ own study).

For the first scenario, parameters for the accuracy evaluation were determined on the basis of 122 points on control profiles (Table 6, Figure 13a). In this case, average height differences for each analysed method are positive and positive deviations are much larger than the maximum negative height difference. Thus, one may come to the conclusion that points measured with the RTK/RTN GNSS method are located below the points determined using other methods. It is caused by the fact that products obtained from the UAV and TLS do not reflect the actual relief in the case of vegetated areas (they only show to a large extent vegetation over this area), whereas the average difference between data from ALS and GNSS measurements indicates systematic shift in height observations which does not exceed the average error of the ALS point cloud.

In order to evaluate the accuracy of detecting changes, which are caused by the erosion of trails, it was necessary to remove 30 points located outside the paved part of the trail (second calculation scenario) from the analysis. It considerably reduced the average height difference calculated for the UAV and TLS methods (Table 7, Figure 13). Parameters calculated for ALS did not change significantly in comparison to the first calculation scenario. It is important to note that in the case of the UAV, maximum and minimum height differences do not exceed 10 cm and standard deviation is 4.1 cm. The accuracy evaluation parameters for TLS data are less favourable, but they are also within the evaluated measurement accuracy.

Bearing in mind the above results, it may be noticed that the methodology adopted may be used to survey trail sections not covered with vegetation by means of the photogrammetric method based on the UAV with the accuracy of about 50 mm. This is a value of about three GSD and can be identified with the accuracy of UAV-based photogrammetric products in alpine areas, for linear objects, where the relative height differences exceed even 1000 m. It should be emphasized that the results obtained can be considered satisfactory, in particular in the light of the research results published so far [36,38,55]. Although obtaining accurate point clouds with UAVs seems to be easy to do nowadays, the results published so far refer to surface objects located in the alpine areas (landslides in particular) or elongated objects with a slight inclination along their axis. This study has empirically proven that for extensive elongated objects located in alpine areas, obtaining highly-accurate products from UAV-borne photogrammetry is also possible, while maintaining the workflow described.

### 3.2. Possibilities of Using Products Obtained from UAV-Borne Photogrammetry for the Evaluation of Erosion along Hiking Trails 

Castillo et al. [68] compared the accuracy, time, and cost of conventional and new methodologies for determining erosion of elongated objects (in this case gullies). Among others, 3D photo-reconstruction and TLS were compared. Despite its time-consuming character, 3D photo-reconstruction proved to be very competitive in terms of economy of work and quality of results. Unfortunately, this publication does not include UAV-borne photogrammetry. However, UAV imagery is successfully used to build DEM and to determine relief changes [29,34,65,66,67,68,69]. In [65] a UAV was used to collect a time series of high-resolution images over four years at seven epochs to assess landslide dynamics. The authors presented in [65] surface movement maps and the recorded displacement values exceed 7 m. Unfortunately, there is no ground validation for the movement vectors generated. Monitoring of a creek in an alpine environment was presented in [67]. The main result of the study was maps indicating the area-wide elevation differences based on grid files. Maximum elevation differences based on UAV DEMs were up to 2 m. The authors concluded that it is possible to evidently delineate erosion and deposition zones but, in general, results with higher accuracy were expected and the UAV-based approach is not suitable to record steep slopes. The results of our research presented above are in opposition to this conclusion.

Remotely-sensed data from a multi-rotor UAV were also used to estimate soil displacement from timber extraction trails in steep terrain (a vertical difference of over 120 m between the lowest and highest points in modelled terrain) [69]. To calculate the soil displacement volumes, a pre-harvest ALS-derived terrain model was to represent the terrain before the skid trails were constructed. In this study the authors focused only on the cut volume, in total, it was estimated that some 554 m^3^ of earth had been displaced by the construction of skid trails over a cumulative length of 210 m. Although, some check points showed a significant error on the Z value of over 50 cm, the conclusion was that the UAV-based data shows considerable potential for improved environmental management and associated cost reduction. On the other hand the aim of the paper [28] was to monitor gully development by means of the UAV remote sensing. The authors concluded that the presented approach allows to map and measure erosion in very high detail. However, in this case soil erosion was only describe based on generated DTM with very high resolution, no quantitative assessment was provided.

On the other hand, an attempt to detect slight changes in the terrain relief on the basis of data obtained from UAV was described in [29]. In this paper surface changes at the level of single centimetres were detected, but it should be noted that UAV flight altitude during image acquisition was only 8–10 m above ground level.

Taking into account the lack of reliable data on this subject, it was decided to assess the applicability of UAV-based photogrammetry products to evaluation of erosion along hiking trails. To this end, point clouds obtained from the UAV were compared with archival data from ALS for the whole study area. On the basis of the results of these analyses, differential raster maps were generated which allow visual assessment of the degree and reasons for erosive phenomena. It has to be remembered, though, that the ALS archival data is characterised by far lower spatial resolution and, therefore, some spatial objects, such as route beds, boulders, are erosion craters, could have been generalized. Simultaneously, this data is features lower spatial accuracy which can lead to constant shifts in reference to actual data, which has been proved by the measurement of control profiles, as well as statistical analyses (Table 6 and Table 7).

The analyses were performed using CloudCompare software (open source project). For the numeric calculations to be improved, the data from the UAV was subject to resampling where a minimum distance between points was set at 3 cm. In this way, the number of points to be analysed was considerably reduced while the reliability of the analyses undertaken did not decrease. The ALS data was imported in two versions:

- the first one included all classes of points and therefore represented land forms along with the land cover (DSM ALS); and
- the second one included only points classified as ground points, which solely represent landforms (DTM ALS).

Subsequently, for the results of comparison to be as reliable as possible, TIN (triangulated irregular network) models were prepared for ALS data. It enabled the calculation of the distances between the clouds of points along normal vectors to the generated triangles and, therefore, the information was obtained about the direction of changes in the land form (decrease/increase). Moreover, the influence of the survey data density on the accuracy of determined distances was eliminated.

As a result of the analysis, for each point from UAV point clouds, a value of deviation from DSM ALS and/or DTM ALS was obtained which derives from the comparison of archival and current data. However, because browsing the point data results requires appropriate software, as well as a lot of RAM, the results obtained were presented as a raster dataset which enables convenient browsing of materials in the GIS environment. To this end, based on UAV point clouds and calculated differences, rasters with a resolution of 5 cm were generated.

Considering the characteristics of the compared data, the proper interpretation of the obtained maps of differences should consist of locating local disturbances of differences and comparing them with the image on the high-resolution orthophotomap. It enables establishing what causes these variations or to note that they are related to measurement errors (e.g., people on the trail during measurements).

**Figure 14:**
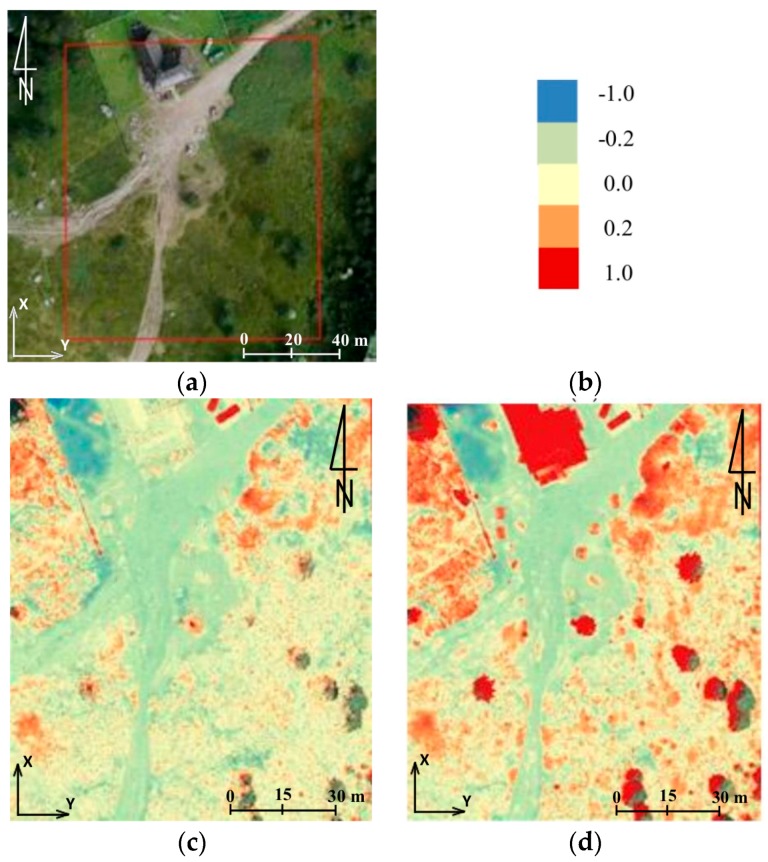
The mountain shelter in Hala Kondratowa: (**a**) the study area, (**b**) colour scale (the units given in metres), (**c**) the results of comparison of DSM UAV–DSM ALS, and (**d**) the results of comparison of DSM UAV–DTM ALS (source: authors’ own study).

Below, several hypothetical study areas are presented. They illustrate possible interpretations of the acquired materials. In all of the examples shown below the same colour scale has been used (Figure 14b). It must be emphasised that data from ALS was treated as referential data and, therefore, negative values (blue) prove the decrease within the period between measurement series; whereas positive values (red) prove the increase (accumulation) or the occurrence of objects in the UAV data that do not occur in the ALS data. In the area presented in Figure 14a, on the basis of differences between DSM UAV and DSM ALS identified for the roofing of the mountain shelter (Figure 14c) a relative accuracy of both sets of data can be calculated. Assuming that between the measurement periods the roof of the shelter was not subject to any displacements, this accuracy oscillates between −20 cm and +10 cm.

Another aspect which can be observed in the discussed example are the places marked with a distinct orange-red colour which, in the case of the DSM UAV–DSM ALS comparison, prove the increase in vegetation. However, this increase can depend on the period in which measurements were taken while, in case of the DSM UAV–DTM ALS comparison, it proves the occurrence of medium and high vegetation (Figure 14d).

When analysing the trail area, one may notice immediately the trail bed and small relative differences of colours, which indicate erosion (more blue ones) or accumulation (light yellow-orange ones). However, in the above-mentioned area such changes are insignificant.

**Figure 15:**
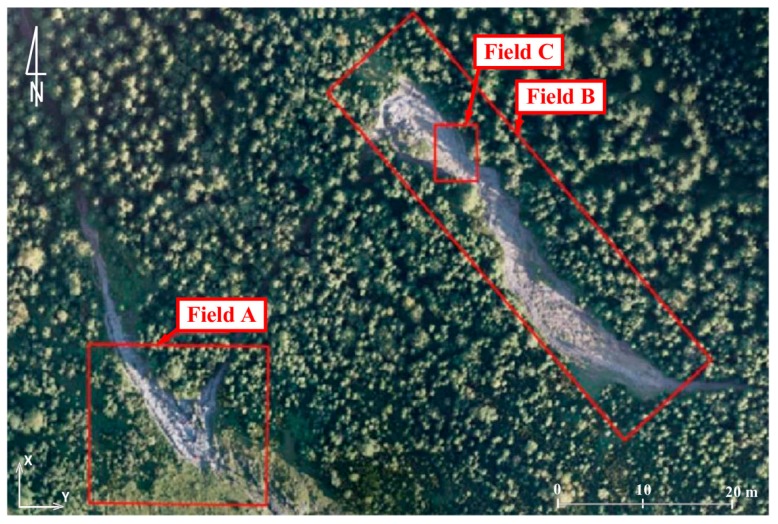
Test field: yellow trail in the area between Jaworzynka and Siodłowa Perć (source: authors’ own study).

**Figure 16:**
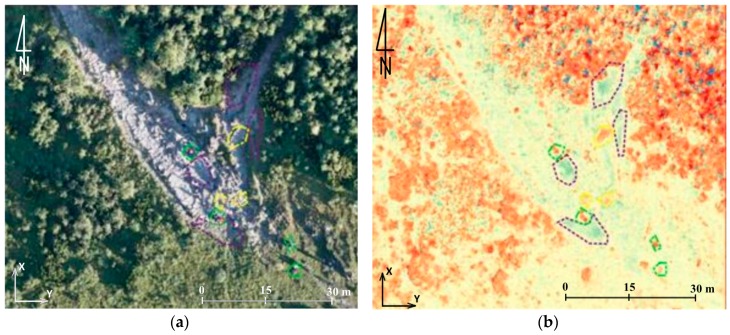
Field A: (**a**) ortophotomap, and (**b**) the results of comparison of DSM UAV–DSM ALS (source: authors’ own study).

**Figure 17:**
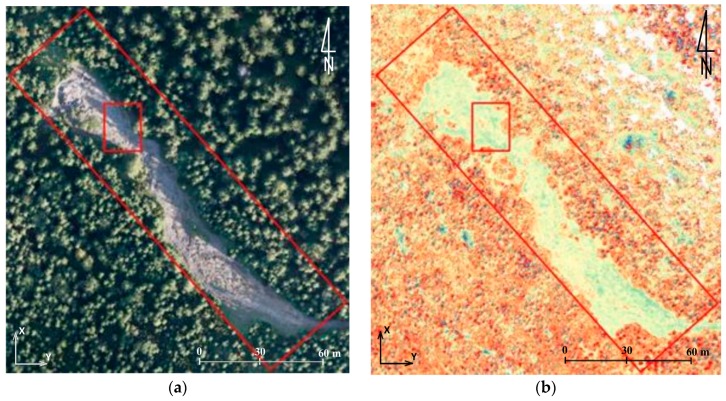
Field B: (**a**) ortophotomap, and (**b**) the results of comparison of DSM UAV–DSM ALS (source: authors’ own study).

**Figure 18:**
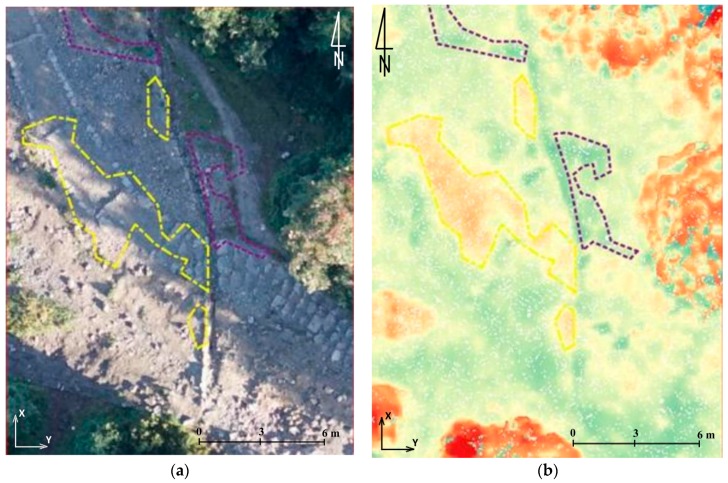
Field C: (**a**) ortophotomap, and (**b**) the results of comparison of DSM UAV–DSM ALS (source: authors’ own study).

Another comparison area was the yellow trail in the area of Jaworzynka-Siodłowa Perć (Figure 15, Figure 16, Figure 17 and Figure 18) which shows how the collected materials can be interpreted. In the images presented below, the following changes occurring on the trail can be observed:

- violet line: places of increased erosion;
- yellow line: places of accumulation; and
- green line: errors in measurement data resulting from the presence of people on the trail during the measurement.

**Figure 19:**
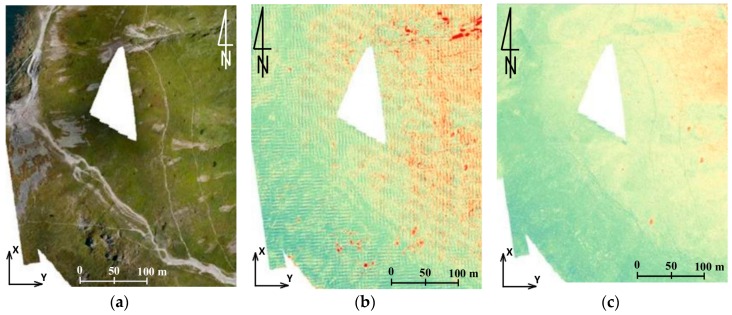
Kopa Kondracka: (**a**) the comparison area on the orthophotomap, (**b**) the result of the comparisons based on raster layers, and (**c**) the results of comparisons based on clouds of points (source: authors’ own study).

Considering the sustainably lower resolution and pixel size in the archival data (35 cm), the analyses were conducted on the basis of clouds of points. The example presented below (Figure 19) illustrates the results obtained from the analysis based on the comparison of raster layers. On its basis, it can be ascertained that conducting such studies for the data of various resolution is not fully reliable and gives limited possibilities of interpretation (significant disturbances of results due to the resolution of data and the mountain slope inclination). Simultaneously, results based on the direct comparison of the clouds of points were presented for the same region. It has to be highlighted, though, that operation on the clouds of points requires increased processing power and can be impossible for vast areas.

### 3.3. The Examples of Interpretation of Raster Images Obtained from a UAV

The photogrammetric methods are some of the most popular sources of data used in work related to forest administration. They started to be used in 1887 when the first photogrammetric photographs were taken with a camera lifted by a balloon. The photographs were later applied for settling a forest [70]. Around 1890 the first successful attempts of using terrestrial photogrammetry for mapping forest stands were made [71]. A significant breakthrough in the development of photogrammetry for the needs of the analysis of forest stands came along with the development of plane aviation. One of the first photogrammetric flights used by forestry took place in Bavaria in 1924 [72].

Currently, in the majority of cases, laser scanning measurements are performed concurrently with taking aerial photos. It is particularly crucial for densely wooded areas where the tree branches make it impossible to take photos from the level of the ground. Considering methods of obtaining more precise data, it is noted that ALS provides accurate information related to forests in vast areas. However, it is often insufficient to define the condition or features of individual trees located within the range of the laser beam. Therefore, TLS is a better method of estimating the condition of a forest stand, especially the density of afforestation [73].

It should also be mentioned that ALS is one of the most expensive methods of assessing the current state of land cover and land forms. Considering the data stored in the generally accessible public databases, it has to be remembered that the time in which the data was obtained might not correlate with the timing assumptions of the conducted project. Therefore, the potential of UAVs needs to be noted as these devices can supply information about the measured area in the chosen time, provided that weather conditions are favourable. An additional advantage is the scale of the photographs taken. One study [74] indicates that the scale of aerial photographs might not be sufficient for the needs of identifying individual tree tops. In the case of using UAV units this problem is minimised by the adequate selection of parameters of the mission which enables obtaining a high resolution of up to 1 cm.

The materials produced during the study can be used for analyses concerning the land cover, such as monitoring the anthropogenic changes of the environment or the extraction of tree tops [75]. Although it is common practice to use the infrared band during vegetation analysis, and it provides very satisfactory results [76,77], the objective of this article is to show the visible range data application in this case. The data presented in the form of a high-resolution orthophotomap allow for the supervised and unsupervised classification aimed at obtaining the land cover maps.

Methods of processing raster information are subject to modifications in relation to the clustering procedures and classifications compiled for satellite depiction [76]. Differences mostly in geometric, radiometric, and spectral resolution lead to a different approach to the processing of georasters. The supervised classification of the nearest neighbourhood was chosen considering the main aim of the classification, which is the separation of vegetated areas from bare ground and bedrock. However, the training sample needs to be selected every time when the areas are photographed in different times of a day or on different days. The changing morphology of the land forms is also significant [78].

**Figure 20:**
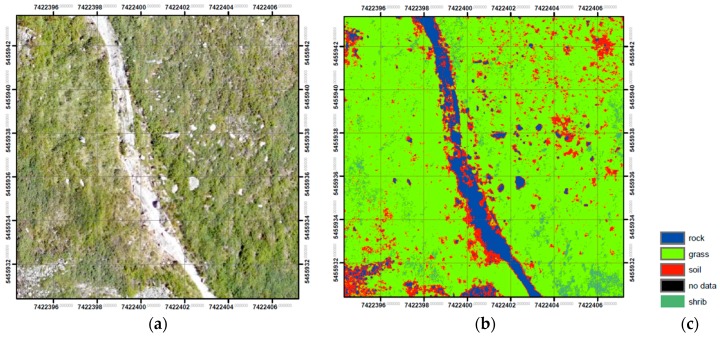
(**a**) An extract from the orthophotomap including the area of Kopa Kondracka (the area undergoing the floral succession); (**b**) the land cover map created by means of supervised classification; (**c**) legend (source: authors’ own study).

In the conducted analyses, training samples included from 2% to 5% of all pixels in the classes. The thematic map was created as a result of the division of the orthophotomap into five classes (rocks, grass and low flora, bushes, soils, lack of data). In the areas included in the research limestone and granite rocks are dominant, which are characterised by high spectral response within the range of visible light [79]. This simplifies the classification. By creating the land cover map (Figure 20) it is possible to conduct all of the quantitative analyses concerning relations between vegetation, bedrock, and soils. Defining the floral succession is possible due to the statistical comparison of the classified data. Another method which provides similar results is the calculation of greenness index (GI) [76]. In the studied case it was, however, less effective than the supervised classification.

**Figure 21:**
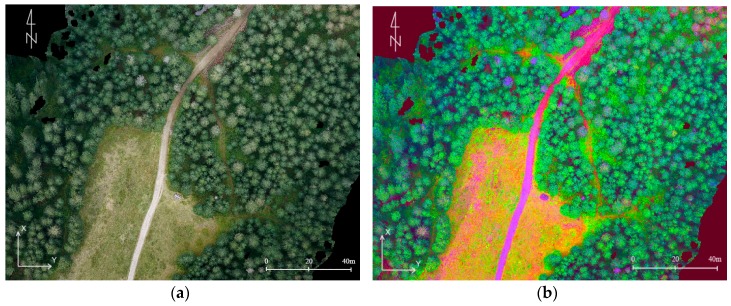
(**a**) A fragment of the orthophotomap with the tourist trail between Dolina Chochołowska and Trzydniowiański Wierch; and (**b**) decorrelation stretching conducted on the basis of the analysis of the main components (source: authors’ own study).

**Figure 22:**
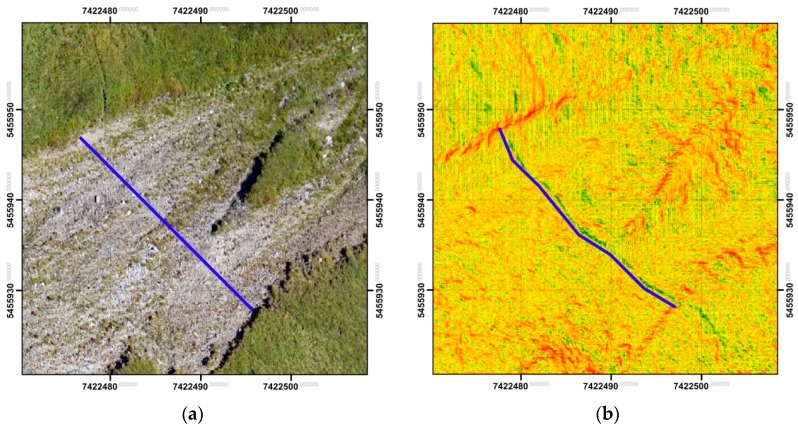
Rock debris: (**a**) orthoimage covering the invisible path; and (**b**) a map of slope allowing the detection of the trail (source: authors’ own study).

A method supporting the identification of objects is decorrelation stretching based on PCA (principal component analysis). The decorrelation stretch is a process to enhance the colour separation. The algorithm starts from each band centre and standardizes, then a covariance matrix is computed. To convert the data into the PCA space, it is necessary to multiply the RGB data by covariance matrix eigenvectors. To produce the decorrelation stretched image, the principal component image is modified by the linear transformation. Subsequently, then the inverse PCA transformation is conducted [80,81]. The application of the histogram decorrelation stretching facilitated the identification of trees that succumb to diseases or atrophy (Figure 21). The sole analysis of components allows the separation of tree tops. Decorrelation stretching enables efficient processing of large amounts of data and it does not take a great deal of time. The analysis of the DSM is a significant research tool. An analysis of slopes calculated on the basis of high-resolution DSM enables the detection of the hiking trail location which cannot be identified on a raw or pre-processed orthophotomap (Figure 22).

## 4. Conclusions

This article describes, in detail, the process of spatial data acquisition by means of a UAV in difficult alpine areas. Moreover, it presents various possibilities of survey data analysis which are aimed at the assessment of erosion along hiking trails, as well as at studying land cover changes, particularly the floral succession in the immediate vicinity of hiking trails.

The area in which the research was conducted is demanding and imposes the necessity of precise planning of the UAV flights, as well as positioning control points with regard to a great number of factors. At this stage it is particularly important to focus on the land forms and forest cover.

Basic products resulting from UAV flights (point clouds, DTMs, orthophotomosaics) make a valuable source material for advanced spatial analyses. Their accuracy assessment parameters, calculated by comparing them with the results of measurements conducted with GNSS and TLS methods, are satisfactory. The methodology adopted enabled the surveying of trails in alpine areas not covered with vegetation by means of UAV imagery with an accuracy of about 50 mm. Although similar accuracies have been repeatedly confirmed in various published studies, they never referred to large, elongated objects with a very high inclination along their axis. It should be noted that the trails subjected to examination were from 1 m to 15 m wide and a vertical difference between the lowest and highest points in modelled terrain exceeded 1000 m.

Advanced analyses consisting in the comparison of data from various measurement periods were conducted in the next part of the study. It has to be emphasised that some of these analyses were made by comparing the obtained materials with historical data that features lower accuracy and spatial resolution. Despite this, the comparisons of point clouds facilitate the interpretation of changes occurring along the hiking trails. By analysing the generated differential maps, it is possible to define places with locally disturbed values of differences. However, such places should be additionally analysed on high-resolution orthophotomaps in order to eliminate factors which can distort the results of analyses, such as the increase in vegetation, or the presence of people on the trail during measurements. The application of various methods of digital analysis of images allows for automatic detection of phenomena and relations which are invisible for an observer, while being more efficient than manual data processing.

The presented studies do not exhaust the potential of analyses or their interpretations. Additionally, it is worth emphasizing that the most effective method would be to compare the data of similar accuracy and resolution parameters conducted in a similar period of the year. Therefore, it seems justifiable to conduct further measurements using the UAV in the following years. The data obtained in such a way would certainly result in the increase in the reliability of defining even small (a few centimetres long) and short-term changes in the morphology of the terrain or forest succession. By means of this, it will be possible to define the effectiveness of protective actions taken by the TNP. This is because of the high density and high accuracy of the collected data. It also seems justified to examine the possibilities of limiting the number of necessary terrestrial control points (without losing the spatial accuracy) which, in such a differentiated and spatially difficult survey area, is going to allow for the optimisation of the data acquisition time.

## Figures and Tables

**Table 1:** Summary of the number of images, control and check points and flight parameters for each photogrammetric mission.

Mission	Number of Images	Real Flight Height above Ground Level (m)	Maximum Height Differences between Waypoints within One Mission (m)	Number of Points of the Photogrammetric Control	
				Control Points	Check Points
Czerwone Wierchy (13 missions)	2889	100–150	180–840	143	113
Kobylarz (three missions)	351	100	230–365	16	10
Grzes (five missions)	783	150	250–315	29	17
Trzydniowianski Wierch (nine missions)	1806	100–150	200–410	75	48
Jaworzynka-Siodłowa Perć (two missions)	254	150	230	9	7
Polana Olczyska (two missions)	488	150	90	11	8
Przysłop Miętusi (one mission)	172	100	90	9	5
Wielka Polana (two missions)	368	100	140	12	5

**Table 2:** The initial root mean square errors of control points and check points.

Mission		*m*_x_ (mm)	*m*_y_ (mm)	*m*_h_ (mm)	*m*_xy_ (mm)	*m*_xyh_ (mm)
Czerwone Wierchy (13 missions)	control points	16	13	17	20	26
	check points	19	17	29	25	38
Kobylarz (three missions)	control points	56	57	34	80	88
	check points	68	50	63	85	102
Grzes (five missions)	control points	8	9	14	11	18
	check points	15	15	22	21	30
Trzydniowianski Wierch (nine missions)	control points	16	18	23	24	33
	check points	15	18	31	23	39
Jaworzynka-Siodłowa Perć (two missions)	control points	14	22	30	26	40
	check points	18	21	37	28	47
Polana Olczyska (two missions)	control points	12	17	26	21	33
	check points	20	17	23	26	35
Przysłop Miętusi (one mission)	control points	17	5	10	18	20
	check points	15	6	20	16	26
Wielka Polana (two missions)	control points	18	15	10	23	26
	check points	18	20	96	27	100
**Average**	**control points**	**24**	**25**	**22**	**34**	**41**
	**check points**	**29**	**24**	**47**	**37**	**60**

**Table 3:** Final mean square errors of points coordinates of the photogrammetric control.

Mission	*m*_x_ (mm)	*m*_y_ (mm)	*m*_h_ (mm)	*m*_xy_ (mm)	*m*_xyh_ (mm)
Czerwone Wierchy (13 missions)	28	25	37	38	53
Kobylarz (three missions)	56	63	29	84	90
Grzes (five missions)	16	19	25	25	36
Trzydniowianski Wierch (nine missions)	18	21	30	27	41
Jaworzynka-Siodłowa Perć (two missions)	13	20	31	24	39
Polana Olczyska (two missions)	18	18	28	26	38
Przysłop Miętusi (one missions)	22	8	24	23	34
Wielka Polana (two missions)	35	24	38	42	56
**Avarage**	**29**	**29**	**31**	**41**	**51**

**Table 4:** The basic parameters of final products generated on the basis of photographs.

Mission	Number of Tie Points	Number of Points in the Point Cloud	Pixel Size for the Orthophotomap	DEM Mesh Size
Czerwone Wierchy (13 missions)	2,012,363	360,257,300	15–20 mm	25–35 mm
Kobylarz (three missions)	356,169	428,923,000	15 mm	25–30 mm
Grzes (five missions)	751,761	799,493,000	20 mm	30–40 mm
Trzydniowianski Wierch (nine missions)	1,565,467	1,888,167,000	15–20 mm	30–40 mm
Jaworzynka-Siodłowa Perć (two missions)	187,664	253,844,000	20 mm	37 mm
Polana Olczyska (two missions)	350,874	312,586,000	20 mm	35 mm
Przysłop Miętusi (one missions)	117,043	151,814,000	15 mm	27 mm
Wielka Polana (two missions)	371,399	417,357,000	15 mm	29 mm

**Table 5:** Results of comparing the point cloud from TLS with the point cloud and the mesh model from the UAV.

Test Field	Reference Object	Compared Object	Average Difference	Standard Deviation
first	UAV point cloud	thinned point cloud from TLS	0.050 m	0.058 m
	UAV mesh	thinned point cloud from TLS	0.048 m	0.054 m
	UAV point cloud	point cloud from TLS	0.042 m	0.044 m
	UAV mesh	point cloud from TLS	0.043 m	0.044 m
second	UAV point cloud	point cloud from TLS	0.062 m	0.013 m

**Table 6:** Accuracy evaluation parameters of acquired materials for the first calculation scenario.

Parameter	GNSS–ALS	GNSS–UAV	GNSS–TLS
Average difference	0.174 m	0.043 m	0.074 m
Maximum difference	0.425 m	0.399 m	0.573 m
Minimum difference	−0.154 m	−0.100 m	−0.329 m
Standard deviation	0.115 m	0.132 m	0.139 m

**Table 7:** Accuracy evaluation parameters of acquired materials for the second calculation scenario.

Parameter	GNSS–ALS	GNSS–UAV	GNSS–TLS
Average difference	0.139 m	−0.027 m	0.016 m
Maximum difference	0.416 m	0.081 m	0.239 m
Minimum difference	−0.154 m	−0.100 m	−0.070 m
Standard deviation	0.099 m	0.041 m	0.060 m
